# Micro energy harvesting for IoT platform: Review analysis toward future research opportunities

**DOI:** 10.1016/j.heliyon.2024.e27778

**Published:** 2024-03-12

**Authors:** Mahidur R. Sarker, Amna Riaz, M.S. Hossain Lipu, Mohamad Hanif Md Saad, Mohammad Nazir Ahmad, Rabiah Abdul Kadir, José Luis Olazagoitia

**Affiliations:** a Institute of Visual Informatics, Universiti Kebangsaan Malaysia, Bangi, 43600, Selangor, Malaysia; b Universidad de Diseño, Innovación y Tecnología, UDIT, Av. Alfonso XIII, 97, 28016 Madrid, Spain; c Department of Electrical Engineering, Bahauddin Zakariya University, Punjab, Pakistan; d Department of Electrical and Electronic Engineering, Green University of Bangladesh, Dhaka, 1207, Bangladesh; e Department of Mechanical Engineering, Faculty of Engineering and Built Environment, Universiti Kebangsaan Malaysia, Bangi, 43600, Selangor, Malaysia

**Keywords:** Internet of things, Micro energy harvesting, Low-cost sensors, Ultra-low power, Low power applications

## Abstract

Micro-energy harvesting (MEH) is a technology of renewable power generation which is a key technology for hosting the future low-powered electronic devices for wireless sensor networks (WSNs) and, the Internet of Things (IoT). Recent technological advancements have given rise to several resources and technologies that are boosting particular facets of society. Many researchers are now interested in studying MEH systems for ultra-low power IoT sensors and WSNs. A comprehensive study of IoT will help to manage a single MEH as a power source for multiple WSNs. The popular database from Scopus was used in this study to perform a review analysis of the MEH system for ultra-low power IoT sensors. All relevant and important literature studies published in this field were statistically analysed using a review analysis method by VOSviewer software, and research gaps, challenges and recommendations of this field were investigated. The findings of the study indicate that there has been an increasing number of literature studies published on the subject of MEH systems for IoT platforms throughout time, particularly from 2013 to 2023. The results demonstrate that 67% of manuscripts highlight problem-solving, modelling and technical overview, simulation, experimental setup and prototype. In observation, 27% of papers are based on bibliometric analysis, systematic review, survey, review and based on case study, and 2% of conference manuscripts are based on modelling, simulation, and review analysis. The top-cited articles are published in 5 different countries and 9 publishers including IEEE 51%, Elsevier 16%, MDPI 10% and others. In addition, several MEH system-related problems and challenges are noted to identify current limitations and research gaps, including technical, modelling, economic, power quality, and environmental concerns. Also, the study offers guidelines and recommendations for the improvement of future MEH technology to increase its energy efficiency, topologies, design, operational performance, and capabilities. This study's detailed information, perceptive analysis, and critical argument are expected to improve MEH research's viable future.

## 1 Introduction

**Fig. 1:**
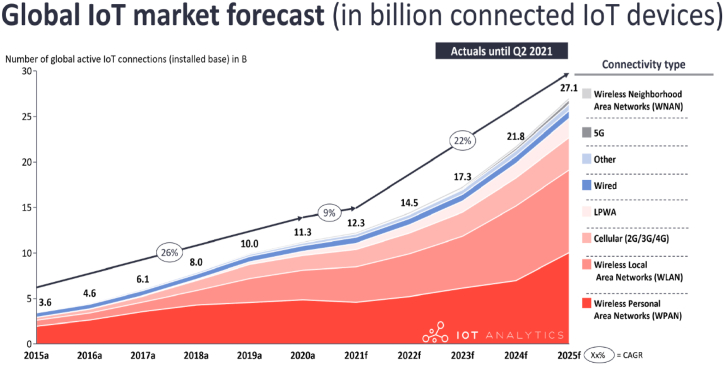
Current and future trends of the global IoT market.

The worldwide deployment satisfying the energy demand, the Internet of Things (IoT) has attracted much attention in recent years. Wireless sensor networks (WSN) and the IoT have played a vital role in our daily lives [1]. However, the limited lifespan of different energy supplies used to power the sensors over time limits the use of IoT and low-power electronic devices. Micro-energy harvesting (MEH) systems need to be improved in terms of efficiency, limitations and the quality of output power. This study reviews various types of ambient energy harvesting devices that can power WSN and IoT devices. There is also discussion of various energy harvesting models that can improve the sustainability of the energy supply needed for IoT sensors. Additionally, the issues must be solved to make IoT-enabled sensors more resilient, dependable, affordable, and energy-efficient. However, no attempts have been made to present the current challenges and the solutions, WSN and IoT devices are currently dealing with. Fig. 1 displays data on IoT devices that are globally connected. There are different kinds of communication devices such as Wireless Network after Next (WNAN), 5G, wired, Low-Power Wide-Area (LPWA), cellular, wireless local-area network (WLAN), wireless personal area network (WPAN) and other components listed in Fig. 1. IoT is rapidly growing, and by 2020, it is predicted that the global market for IoT solutions will reach $7.1 trillion [2]. IoT involves several difficulties, such as setting up and maintaining cloud computing server farms and frequently updating the firmware of millions of smart devices from a maintenance and security standpoint [3].

For many years, IoT technology has been seen as dynamic, adaptive, and interconnected. The phrase IoT was first used in 1999 by Kevin Ashton [4]. IoT is seen as a key technological element of the rapidly developing smart and computer world. Sensing and collection of data and then fast sharing of this data worldwide through the Internet is the basic idea of the creation of an IoT system. Wireless network terminals of a network use IoT-equipped sensors and devices to gather data, information, and statistics. A few examples of the many applications where IoT can be used such as i) embedded systems, ii) security surveillance systems, iii) controllers, iv) transportation systems, v) wearable devices, vi) energy tracking, vii) environmental monitoring systems, viii) fire detection, ix) traffic monitoring, x) smart farming, xi) human body area networking, etc. More than 26 billion IoT devices are anticipated to be online by the end of 2020. This amount could rise to more than $100 billion. Since the power requirements of all the IoT devices are low finding a power source which can fulfil all power requirements without any delay or maintenance is difficult.

The study was carried out using the Web of Science (WoS) database because it's a model that has been previously validated, and it followed the structure and procedures used in other bibliometric-based studies. A review has been conducted based on the top 100 highly cited manuscripts, keywords, abstracts, and methodologies. Bibliometric analysis is one of the greatest tools for understanding topic evaluation over time and identifying the most popular topic in the relevant field [5]. The important characteristics are the frequency of publication by nation, author, impact factor, year, total number of citations, and study type. One of the main bibliometric tools is citation analysis, which has gained popularity as a method of evaluating the influence of journals, specific works of literature, journal editors, or authors.

The literature, as mentioned previously, can provide comprehensive information and guidance on the direction of future studies in the field of MEH systems in IoT and WSN applications, before this bibliometric analysis is important to provide a thorough overview of the subject. In bibliometric analysis, this study intends to provide a thorough assessment of the literature on MEH technologies for IoT applications. 100 of the most highly referenced publications from 2013 to 2023 were taken from the Scopus database for this study. We review the scientific literature using bibliometric evaluations of co-occurrence keyword analysis, bibliometric evaluations of the last five years' worth of citations, study types, journals, subject areas, and affiliations of the authors with the highest profile. The following is a list of this article's ultimate goals:

• Aimed to present a comprehensive study of the most well-known journals, authors, articles, and co-occurrence keywords.
• The efficiency of the MEH system for powering the IoT and WSN applications depends upon power management and low-power devices.
• The reliability of the MEH system relates to the consistency and stability of the IoT devices under variable loads.
• The MEH should be compatible with WSN, IoT devices and power management systems.
• Abundant energy resources reduced the cost of the MEH system. MEH is valuable for powering sensors and devices in industrial settings where wiring or battery replacement may be impractical.
• The literature reviews, research gaps, limitations, and investigations in the area of MEH in IoT applications are highlighted to assist future researchers.
• In addition, the challenges, limitations, and relevant directions for future research are examined in the context of the earlier investigations.
• MEH for IoT devices is an important field of study that aims to solve the problems related to IoT device power requirements.
• EH is particularly beneficial for IoT sensor nodes deployed in WSN for environmental monitoring, agriculture, or smart cities.

## 2 State of art

**Fig. 2:**
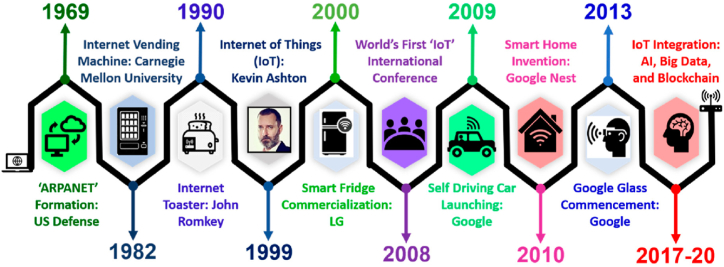
The block diagram of IoT timeline for the last 51 years.

The IoT is an active and vital area of research and can contribute to the advancement of society. Ashton coined the phrase "Internet of Things" in 1998. Accordingly to the authors, the IoT is as important as the Internet was in the early 1990s. In 2001, the MIT Auto-ID Center was a pioneer in presenting an IoT-related concept. The idea and technologies of IoT were formally introduced in a 2005 international telecommunication union (ITU) Internet report. IoT is mainly defined as the IoT, which ideally uses any network and service to link people and things at anytime, anywhere, with anything, and with everyone. IoT sensors are connected and practically every aspect of life, including transportation, safety, home automation, and various wearable technology [6]. Infographics representing the IoT timeline are shown in Fig. 2.

Big data, blockchain-integrated IoT, and artificial intelligence (AI) have all gained in popularity since 2017 [7]. The majority of enclosed systems, including smart speakers, cameras, sensors, meters, etc., are internet-connected and very simple to control. However, a lot of these devices are wireless, sensor-based terminals that are small and difficult to access. Therefore, the only power solution for these IoT nodes is with small batteries. This is a poor solution because installing batteries in small sets are expensive, challenging, and labour-intensive. According to these circumstances, the creation of an MEH system from ambient sources is a viable technique that can assist in resolving issues with the powering of IoT-controlled devices [8].

**Fig. 3:**
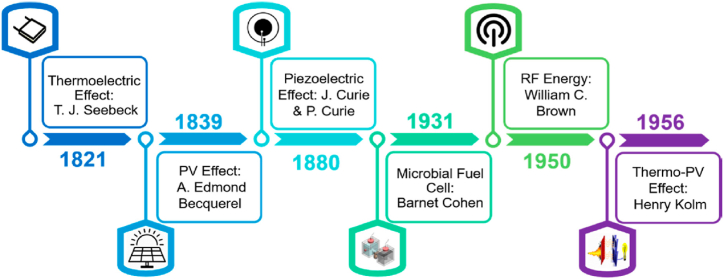
The initial development timeline of MEH system.

The infrastructure supporting the energy market has undergone tremendous development. The IoT sensors currently incorporate the processing power of every energy provider, which could increase their energy consumption [9]. The MEH system should be created to enable the connectivity of IoT platform with a self-powered function to solve the energy crisis [10]. Different researchers have used numerous optimization methods to reduce the amount of power needed to meet current and future energy demands. According to Fig. 3 between 1821 and 1956, the fundamentals of energy sources were defined [11]. Fig. 3 represents the initial timeline for the growth of MEH. The disparities between various energy sources are displayed in Table 1.

**Table 1:** Application of IoT with different ambient sources [12].

Ambient re sources	Features	Transducer	Power Density	Benefits	Drawback	Applications
Thermal energy	Abundant, Linear, relationship of sensor input and output	TEG	40 μW/cm^2^	Clean energy, Constant, efficient.	Low energy, higher cost, and output power depend upon thermal gradient conversion efficiency.	IOT sensors
Wind energy	Abundant, Linear, Relationship of sensor input and output	Wind Turbines	197W/m2	Easily available, low cost	Ideal location in remote sites, turbines produce noise, disturbance for wildlife.	Micro devices
Physical movement of the human body	Human body vibrations, fully controllable	Piezoelectric	2 W	Available	Energy is harvested only with body movement.	Low power electronics
PV	Solar energy	PV Solar cell	6.63W/m2	Clean energy, low cost, low maintenance	High initial cost, space requirement, transportation in installation.	IOT Applications
Vibrational Energy	Abandant, linear	PZT	1000W/cm3	Predictable, reliable, Efficient, low cost	Sometimes cost high, difficult to design small converters.	Ultra-low power sensors
Vehicle Motion	Non-Ambient, Controllable, Partly-predictable	Piezoelectric	332 W/cm2	Low cost	Highly variable output	Resistive load
Human Breathing	Passive power, Non linear	Thermal sensor	1.2m-W/cm	Easily available	–	–
Radio frequency	Abundant, linear	RF sensors	0.1m-W/cm2	Low-cost, environment friendly	Can be harmful for living. power density	Communications

**Fig. 4:**
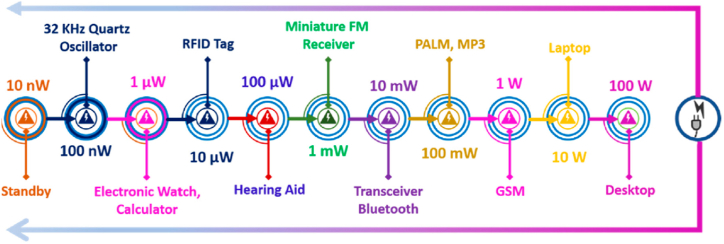
IoT sensors power level in the range of among 10 nW to 100 W.

The power density is an important factor in the application of IoT devices. According to Fig. 4, the IoT devices are divided on the based on their power density. In Fig. 4 shows low power electronic devices in the ranges from 10 nW to 100 W [13]. IoT devices are typically powered by batteries. A significant drawback of battery-powered devices is their finite battery capacity because IoT device communication consumes a significant amount of energy, limiting the period of time that they can work for as long as the battery lasts [14]. For small IoT systems, this battery replacement strategy might work, but it won't work for large IoT systems because it would be exceedingly expensive to maintain and replace billions of batteries [15]. MEH system is a potential solution to this issue. The IoT sensors are powered by this collected electrical energy, which also increases the life of the IoT platform. The harvested energy is very low AC voltage with ripples and load dependent. A signal conditioning circuit for rectification, filtration, and boosting is used to improve the quality of output voltage. The extracted powers are either used directly or stored in an energy storage system for later usage. The efficiency of MEH depends upon the design of the harvester, availability of the abundant energy at the location and power management system.

## 3 Survey and discussion methods

The first step provides the study with a comprehensive discussion covering various aspects, including the significance, technologies, applications, challenges, and future directions of MEH in the context of IoT. The Scopus databases have been used to collect the latest information on highly 100 cited manuscripts in this field of MEH on low-power IoT devices. Among all the manuscripts identified pivotal works and pivotal contributions that have influenced the field of MEH in IoT platforms in terms of emerging trends, technological innovations, validity of results, and practical applicability. This review has discussed how the findings contribute to real-world applications in IoT platforms and assessed the scalability and feasibility of implementing MEH solutions. This article has comprehensively explained and emphasized the contributions of the selected high-citation top 100 manuscripts to the broader field of MEH for IoT application and highlights areas that require further investigation or improvement.

**Fig. 5:**
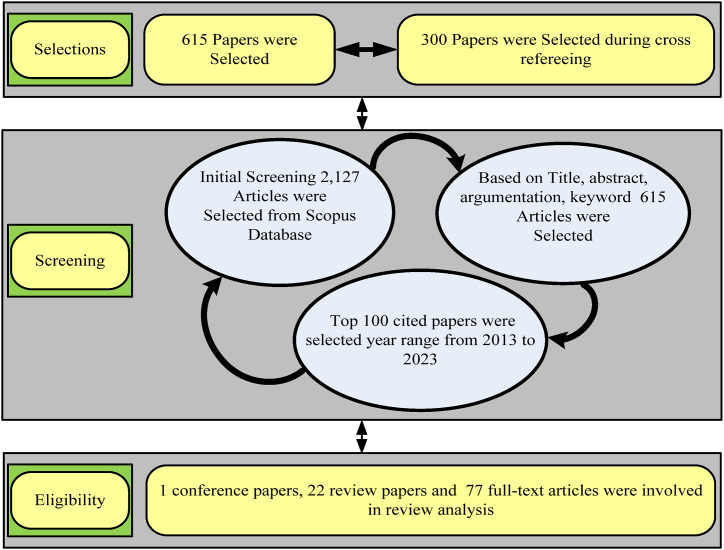
The selection criteria of top 100 cited manuscript.

The main goal is to present an overview of the most recent research in the field of MEH systems for IoT applications and to avoid the limitation of conventional batteries [16]. The authors in Ref. [16] suggested that the use of embedded systems in IoT allows the interoperability of different standards of communication. A quick search was carried out in the Scopus database on the last week of February under the year range of 2013–2023. The Scopus database was searched using the keywords "Micro energy harvesting," "WSN," “battery-less low power devices” and their IoT system integrations. To select manuscripts for the bibliometric study, further filters have been applied by a detailed investigation. One of the reasons for limited resources is the English language filter which has only selected manuscripts in English. The further filtration is based on the highest to lowest cited, title, abstract, keyword and contributions of the manuscript in the relevant field. Fig. 5 shows a schematic diagram that serves as an example of the selection procedure.

The study of Micro Electro Mechanical System (MEMS) technology with integration of diverse harvesting techniques, development of low-power IoT sensors appropriate for micro-devices, and improvements in energy-efficient technologies were all positively addressed [17,18]. Furthermore, by improving energy utilization and reducing network maintenance costs, EH offers both financial and practical benefits. Research interests in MEMS energy harvesters also have been growing quickly, suggesting that the field has made a substantial contribution to the sustainable development of energy alternatives [5]. In the last decade, several studies and reports have been published to expose the advancements in MEMS energy harvesting technology, particularly as they relate to applications in the biomedical, automotive, and consumer electronics industries [19,20]. A wearable sensor node with low-power Bluetooth transmission and solar EH was suggested as part of the implementation of an autonomous wireless body area network (WBAN) by the authors [21]. The authors [22] suggest utilizing a pulse width modulation signal in conjunction with pulse heating to achieve ultra-low power consumption for metal oxide semiconductor gas sensors, along with a matching measuring approach. This author [23] highlights a few important and practical MEMS technology applications for the Internet of Things. A few of the more well-known and often employed MEMS fabrication techniques are reviewed. It focuses on a select few of the most widely used micromachining techniques. MEMS is the technology of the future because of its wide range of possible applications, economic significance, and potential impact on the Internet of Things. The authors in Ref. [24] proposed hybrid energy harvesting and energy-neutral operation with the use of motes fitted with solar energy harvesters. The study in Ref. [25] presents the testing and use of a cost-effective pressure sensor (0–689 kPa) range) for water level monitoring based on MEMS technology and IoT concepts. The sensor performance in terms of accuracy, precision, repeatability, and temperature was investigated in laboratory columns (with constant water level, increasing and decreasing water levels at various rates) and in-situ conditions in an observation bore (with natural groundwater level fluctuations). The results show that the MEMS sensor capable of providing a reliable and adequate monitoring scheme with an accuracy of 0.31% full scale.

MEMS technology offers downsizing, low power consumption, a range of sensors, integration with Complementary Metal-Oxide-Semiconductor (CMOS) technology, affordability, energy harvesting capabilities, wireless communication, and dependability, all laying the groundwork for creating ultra-low power IoT sensors. MEMS helps develop and widely adopt IoT applications across numerous industries.

### 3.1 Manuscript selection criteria

On the base of selection criteria, the most cited articles in the MEH in IoT applications have been selected to find the most useful study resources. The selection criteria for the manuscript from the Scopus database are given below:

• All articles on MEH systems, optimal algorithms, energy storage systems, low-cost sensors, low-power devices, autonomous, and IoT system integration have already been taken into consideration. Topics on the IoT, battery-less low-power devices, autonomous sensors, power management and desalination were among the studies that were excluded.
• For the assessment, highly cited manuscripts that were published between 2013 and 2021 were chosen.
• After first screening 2127 articles were selected from Scopus Database.
• After the second screening 615 manuscripts were selected on the base of title, abstract and keywords.
• In the third screening 100 most cited articles were selected in the relevant field from 2013 to 2023.

### 3.2 Current trend in the research field

**Fig. 6:**
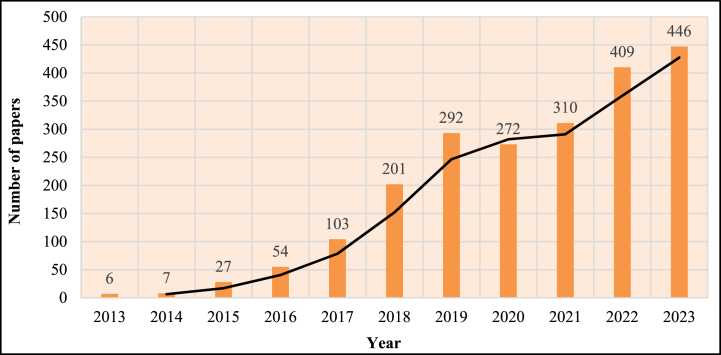
MEH current research trends in IoT applications.

In this study, the current trending and state-of-the-art is associated with EH at the micro level for IoT sensors. Following that, emphasis was placed on stand-alone applications for IoT sensors and WSN, as low-cost and low-powered devices for IoT applications. A Scopus database study of the number of articles published each year in the area of an optimal method in MEH in IoT application is shown in Fig. 6. The figure reveals that the number of articles has significantly increased since 2015, which reflects the expansion of this specific field's study interest. The number of articles published each year from 2015 to 2023 is 2.61%–27.33%, whereas the total of all papers published from 2013 to 2023 is 2127. As a result, it can be concluded that 84.32% of papers were conducted in the final five years, between 2019 and 2023.

### 3.3 Data extraction

Scopus to assess the papers published between the years 2013 and 2023, the Scopus database was used. The following limitations were addressed by the data: i) a list of the most frequently cited articles; ii) study characteristics; iii) the top articles based on citations over the previous five years; iv) the field of study; v) the publisher of the top 100 cited articles; vi) the most prestigious journal and its impact factor; vii) the nation; and viii) the most renowned authors. Finally, an analysis of the Scopus data from the top-most cited articles has been supplied to give a comprehensive understanding of the MEH in the IoT and microelectronic applications.

### 3.4 Bibliometric analysis of the study in terms of top highly cited articles

At first, a total of 2127 articles connected to this topic were found in the Scopus database. According to the analysis, these 100 articles were chosen because their citations range from 43 to 756, for a total of 12,892 citations. Additionally, there have been 8 articles receiving more than 300 of them. On the base of Scopus database article with the most citations was published in IEEE Communication Mag. "Ambient backscatter communications: A contemporary survey" with 542 citations. The second most cited article was published in IEEE Communications Surveys and Tutorials in 2015. The top 100 manuscripts were taken into consideration and are shown in Table 2. Table 2 also highlights the author's name & publication year, keywords, manuscript types, name of journal, publisher, country, and total citations.

**Table 2:** 100 most cited manuscripts in micro energy harvesting systems on low power IoT sensors.

Rank Based on citation	Ref	Author Name & Publication year	Keywords	Manuscript type	Name of Journal	Publisher	Country	Total citation
1	[26]	Liu et al. (2013)	WSN, IoT, Power management	Conference	CCRED	Assoc Computing Machinery	USA	756
2	[27]	Van Huynh et al. (2018)	EH, IoT, WSN, RFID	Article	COMST	IEEE	USA	542
3	[28]	Kamalinejad et al. (2015)	EH, IoT, WSN, Power management	Article	MCOM	IEEE	USA	512
4	[29]	Min et al. (2019)	EH, IoT, Battery, Power management, low-power devices	Article	TVT	IEEE	USA	412
5	[30]	Shi et al. (2020)	EH, IoT, WSN, Battery, Sensors	Review	ADMA	Wiley	Germany	402
6	[31]	Ejaz et al. (2017)	EH, IoT, WSN, Autonomous, Low power, Optimization	Article	MCOM	IEEE	USA	359
7	[32]	Lu et al. (2020)	EH, IoT, Low power, Low cost	Review	NANOEN	Elsevier	Netherlands	334
8	[33]	Narita et al. (2018)	EH, IoT, Low power, WSN, Battery, power management, Low cost, Autonomous	Review	ADEM	Wiley	Germany	331
9	[21]	Wu et al. (2017)	EH, IoT, WSN, Low power, Autonomous	Article	ACCESS	IEEE	USA	288
10	[34]	Martinez et al. (2015)	EH, IoT, Low power, WSN, Low cost	Article	JSEN	IEEE	USA	243
11	[35]	Jin et al. (2020)	EH, IoT, Low power, Low cost	Article	NANOLETT	Amer Chemical Soc	USA	214
12	[36]	Haras et al. (2018)	EH, IoT, Low power electronic, battery, Optimization	Review	NANOEN	Elsevier	Netherlands	214
13	[37]	Pan et al. (2018)	EH, IoT, WSN, Low cost	Article	S	Nature	Germany	200
14	[38]	Mois et al. (2017)	EH, IoT, Low power electronic, WSN, Autonomous	Article	TIM	IEEE	USA	200
15	[39]	Paracha et al. (2019)	EH, IoT, WSN, Autonomous, Low cost	Review	ACCESS	IEEE	USA	185
16	[40]	Landaluce et al. (2020)	EH, IoT, WSN, Wearable RFID, Low cost	Review	S	MDPI	Switzerland	184
17	[41]	Jayakumar et al. (2014)	EH, IoT, Low power devices, battery, storage	Conference	ACM SIGDA	IEEE	USA	179
18	[42]	Ma et al. (2020)	EH, IoT, Sensor, Low-power devices	Review	COMST	IEEE	USA	175
19	[43]	Yan et al. (2018)	EH, IoT, MEMS	Review	JMEMS	IEEE	USA	173
20	[44]	Han et al. (2017)	EH, IoT, WSN, battery	Article	TWC	IEEE	USA	161
21	[45]	Ahmed et al. (2019)	EH, IoT, Energy storage, battery	Review	ADVS	Wiley	USA	159
22	[46]	Prauzek et al. (2018)	EH, IoT, WSN, Storage, battery	Article	Sensors	MDPI	Switzerland	158
23	[47]	Yang et al. (2018)	EH, IoT, Low Power, Power management, Optimization	Article	JIOT	IEEE	USA	154
24	[48]	Akan et al. (2018)	EH, IoT, WSN, battery	Article	JIOT	IEEE	USA	150
25	[49]	Kanan et al. (2018)	EH, IoT, Low power, Storage, battery, Autonomous	Review	AUTCON	Elsevier	Netherlands	149
26	[50]	Min et al. (2019)	EH, IoT, biomedical	Article	JIOT	IEEE	USA	147
27	[51]	Zeadally et al. (2020)	EH, IoT, Low cost, Battery, storage, power management	Review	RSER	Elsevier	England	144
28	[52]	Mori et al. (2018)	EH, IoT, Piezoelectric, WSN, battery	Article	MRS	Springer	Germany	143
29	[53]	Shirvanimoghaddam et al. (2019)	EH, IoT, battery, Piezoelectric, Low power, power management	Article	ACCESS	IEEE	USA	124
30	[54]	Mao et al. (2020)	IoT, AI, Energy efficiency, EH, Optimization	Article	JIOT	IEEE	USA	122
31	[55]	Elahi et al. (2020)	IoT, EH, Battery, Ambient energy	Review	EN	MDPI	Switzerland	118
32	[56]	Maharjan et al. (2018)	Sensor, WSN,Autnomous, EH, IoT, battery, Low cost, Optimization	Article	NANOEN	Elsevier	Netherlands	116
33	[57]	Divakaran et al. (2019)	EH, IoT, Sensors, RF	Review	MMCE	Wiley	USA	115
34	[58]	Gorlatova et al. (2015)	EH, IoT, Low power network, low-power, WSN	Article	JSAC	IEEE	USA	114
35	[59]	Nguyen et al. (2018)	EH, IoT, Energy back-off	Article	TGCN	IEEE	USA	111
36	[60]	Muncuk et al. (2018)	EH, IoT, battery, RF, power management	Article	JIOT	IEEE	USA	110
37	[61]	Liu et al. (2019)	EH, IoT devices, Low-power devices, storage	Article	MCOM	IEEE	USA	110
38	[62]	Annapureddy et al. (2017)	EH, IoT, Low power devices, battery, Autonomous	Article	C	Royal Society of Chemistry	England	109
39	[63]	Sodhro et al. (2018)	Battery lifecycle, Optimization, IoT, power management	Article	FUTURE	Elsevier	Netherlands	107
40	[64]	Du et al. (2017)	EH, IoT, Piezoelectric, Autonomous, Low-power devices, power management	Article	JSSC	IEEE	USA	107
41	[65]	Kang et al. (2018)	EH, IoT, low power devices, power management, Optimization	Article	TWC	IEEE	USA	105
42	[66]	Aslam et al. (2020)	IoT, Sensors, EH, PV, Autonomous	Review	SOLENER	Elsevier	England	105
43	[67]	Sherazi et al. (2018)	EH, IoT, WSN, storage	Article	ADHOC	Elsevier	Netherlands	97
44	[68]	Fan et al. (2020)	IoT, Low power devices, WSN, EH, battery, Power management	Article	NANOEN	Elsevier	Netherlands	97
45	[69]	Sanislav et al. (2021)	EH, IoT, Low cost, battery, WSN, power management	Article	ACCESS	IEEE	USA	95
46	[70]	Choi et al. (2018)	EH, IoT, Low power, WSN, power management	Article	JIOT	IEEE	USA	93
47	[71]	Wei et al. (2019)	EH, IoT devices, Low power devices, battery	Article	JIOT	IEEE	USA	93
48	[72]	Lee et al. (2019)	EH, IoT, Piezoelectric, Storage, power management	Review	STAM	Taylor and Francis	England	90
49	[73]	Liu et al. (2015)	EH, IoT, MPPT, power management	Article	TVLSI	IEEE	USA	87
50	[74]	Lazaro et al. (2018)	EH, IoT, Low cost devices, battery	Review	S	MDPI	Switzerland	87
51	[75]	Shafique et al. (2018)	EH, IoT devices, Low cost & power, battery	Article	ACCESS	IEEE	USA	86
52	[76]	Sadowski et al. (2020)	IoT, WSN, EH, power management	Article	COMPAG	Elsevier	England	86
53	[77]	Kang et al. (2018)	Magnetoelectric, IoT, EH, WSN	Article	AENM	Wiley	Germany	73
54	[78]	Lim et al. (2019)	IoT, EH, Low power devices, WSN, storage	Article	C	Royal Society of Chemistry	USA	84
55	[79]	Din et al. (2019)	EH, IoT, WSN, power management, Autonomous	Article	FUTURE	Elsevier	Netherlands	83
56	[80]	Wu et al. (2018)	WSN, EH, IoT	Article	ACCESS	IEEE	USA	82
57	[81]	Carreon et al. (2016)	Converter, Autonomous, EH, IoT, WSN, Power management	Article	JSSC	IEEE	USA	82
58	[82]	Zhang et al. (2020)	EH, IoT, Optimization	Article	TMC	IEEE	USA	82
59	[83]	Gurjar et al. (2019)	IoT, RF, WSN, Low power, EH	Article	JIOT	IEEE	USA	79
60	[84]	La Rosa et al. (2019)	IoT devices, WSN, battery, EH, Low cost, power management	Article	S	MDPI	Switzerland	79
61	[85]	Zabek et al. (2017)	EH, IoT, PVDF, Piezoelectric, Storage	Article	ACSAMI	American Chemical Society	USA	78
62	[86]	Aslam et al. (2018)	EH, IoT, WSN, Power management	Article	JIOT	IEEE	USA	76
63	[87]	Huang et al. (2018)	EH, IoT, WSN, Low power devices	Article	JIOT	IEEE	USA	76
64	[88]	Maharjan et al. (2020)	Low power; EH, Low cost devices, WSN, battery	Article	AENM	Wiley	Germany	76
65	[89]	Iannacci et al. (2018)	EH, IoT, IoE, RF, WSN	Review	SNA	Elsevier	Switzerland	75
66	[90]	Singh et al. (2021)	EH, IoT, WSN, battery, Autonomous, power management, Low cost	Review	RE	Wiley	USA	75
67	[91]	Sun et al. (2019)	EH, IoT, Sensors, Low power devices, WSN, Battery, Optimization	Article	NANOEN	Elsevier	Netherlands	74
68	[92]	Abella et al. (2019)	IoT, EH, Low power sensor, Autonomous, WSN, Storage, Low cost	Article	JSEN	IEEE	USA	73
69	[93]	Hou et al. (2018)	IoT devices, WSN, EH, Low cost, power management	Article	JIOT	IEEE	USA	73
70	[94]	Saleem et al. (2018)	EH, IoT, Controller, Low power, Power management	Article	TII	IEEE	USA	71
71	[95]	Mohd et al. (2018)	IoT, EH, Low power devices, battery, Optimization	Article	ACCESS	IEEE	USA	69
72	[96]	Ghosh et al. (2020)	EH, IoT, Low cost devices, WSN	Article	Acssuschemeng	American Chemical Society	USA	69
73	[97]	Lau et al. (2019)	IoT, EH, Storage, Autonomous, Power management	Review	MTENER	Elsevier	England	68
74	[98]	Qian et al. (2019)	EH, IoT, Algorithm	Article	JIOT	IEEE	USA	67
75	[99]	Tang et al. (2018)	EH, IoT, WSN, Storage, battery	Article	S	MDPI	Switzerland	66
76	[100]	Jeong et al. (2019)	IoT, Piezoelectric EH, Low cost devices	Article	C	Royal Society of Chemistry	England	66
77	[101]	Adegbija et al. (2018)	IoT devices, EH, Low power, Low cost, Optimization	Article	TCAD	IEEE	USA	65
78	[102]	AlRikabi et al. (2019)	Low power devices, EH, WSN, Optimization	Article	IJET	Kassel University Press GmbH	Germany	64
79	[103]	Saraereh et al. (2020)	EH, IoT devices, battery, TEG, WSN, Autonomous	Article	Sensors	MDPI	Switzerland	63
80	[104]	Alsharif et al. (2019)	EH, IoT, WSN, power management	Review	SYM	MDPI	Switzerland	63
81	[105]	Ullah et al. (2022)	EH, IoT, Low power, WSN	Review	ACCESS	IEEE	USA	63
82	[106]	Wu et al. (2018)	EH, IoT, WSN, Autonomous	Article	ACCESS	IEEE	USA	62
83	[107]	Guo et al. (2016)	EH, IoT, Optimization, Low power devices, WSN, battery	Article	MCOM	IEEE	USA	61
84	[108]	Li et al. (2020)	EH, IoT, low power, WSN	Article	NANOEN	Elsevier	Netherlands	61
85	[109]	Chen et al. (2023)	Hybrid energy supply, IoT, optimization	Article	TST	Tsinghua univ press	China	59
86	[110]	Gupta et al. (2017)	EH, IoT devices, Low power, Optimization	Article	ACCESS	IEEE	USA	59
87	[111]	Loss et al. (2016)	IoT, EH, WSN, battery	Article	S	MDPI	Switzerland	59
88	[112]	Jameel et al. (2019)	Smart networking, IoT, EH, battery, Power management	Article	EJWCN	Springer International Publishing	USA	57
89	[113]	Maurya et al. (2018)	IoT, EH, Piezoelectric, WSN, Power management	Review	JMR	Cambridge University Press	Germany	56
90	[114]	Kim et al. (2020)	EH, IoT, Battery, storage	Article	C	Royal Society of Chemistry	England	56
91	[115]	Liu et al. (2017)	IoT devices, EH, WSN, battery	Article	TWC	IEEE	USA	55
92	[116]	Lin et al. (2022)	EH, IoT, blockchain	Article	JIOT	IEEE	USA	54
93	[117]	Sun et al. (2018)	EH, IoT, storage, devices, power management	Review	S	Springer	Germany	53
94	[118]	Rauniyar et al. (2019)	EH, IoT, low power sensors, Optimization	Article	JSEN	IEEE	USA	50
95	[119]	Joris et al. (2019)	EH, IoT, WSN, low power sensors, Autonomous	Article	LSENS	IEEE	USA	50
96	[120]	Na et al. (2019)	IoT, SWIPT, EH, power, battery, management	Article	JIOT	IEEE	USA	48
97	[121]	Correia et al. (2019)	IoT, RF, WSN, battery, Low cost	Article	TMTT	IEEE	USA	48
98	[122]	Ozger et al. (2018)	EH, IoT, battery, WSN, Smart Grid, power management	Article	S	Springer	USA	44
99	[123]	Kantareddy et al. (2019)	EH, IoT, WSN, battery less sensors, Low cost	Article	JIOT	IEEE	USA	43
100	[124]	Somkuwar et al. (2018)	Vibration, EH, IoT	Article	S	Springer	USA	43

Simultaneous wireless information and power transfer = SWIPT.

Thermoelectric generator (TEG).

This paper constructively discussed in terms of the advancements in energy-efficient technologies, integration of various harvesting methods, exploration of novel materials for energy conversion and low-power IoT sensors which are suitable for micro-devices [28,29,31]. The work in Ref. [29] discussed how sensors lifespan can be increased by EH technology which also occasionally eliminates the need for batteries. The authors also addressed EH provides practical and financial benefits by optimizing energy use and lowering network maintenance expenses. In Ref. [21] the authors proposed to implement an autonomous WBAN, a wearable sensor node with low-power Bluetooth transmission and solar energy harvesting. The advantage is when the patient spends a small amount of time outside and long-term continuous medical monitoring based on WBAN is feasible, as demonstrated by the suggested system with solar EH. Nevertheless, a drawback of the system was that it failed to address the system's overall efficiency when calculating loss during hardware implementation. The study in Ref. [53] highlighted piezoelectric EH in particular as one of the most promising approaches to powering IoT devices. In Ref. [57], the authors reported the main issue to implement the IoT devices is the power supply. Therefore, in this study, the RFEH technique has been proposed to overcome the problem, where solar is not available. In Ref. [64], the authors proposed a synchronized switch harvesting (SSH) technique using 10 mH inductor values to power up the self-power IoT devices. The outcome shows the prototype size 0.35- μm CMOS process, that 9.7 × performance improvement with an 80% efficiency compared to a conventional full-bridge rectifier. The work reported in Ref. [68] presented a hybrid Triboelectric and electromagnetic generators EH method to power up IoT devices which measure wind speed. The model produces 15 m/s wind speed with voltage levels 416 V and 63.2 V, on the other hand when the wind speed is 9 m/s with the power levels 0.36 mW and 18.6 mW, corresponding. In Ref. [81], the authors reported a DC-DC boost converter prototype using a 180 nm CMOS process, to raise the power outcome 1.75 mW input with an efficiency of 57%. The selected technologies and micro energy harvesting techniques are state-of-the-art models that perform better than the contemporary techniques in terms of strong computation capability, improved generalization performance, enhanced efficiency and precision. The analysis covers each MEH technique, optimization parameters for the EH controller and low-power IoT devices strategy's objectives, advantages, and drawbacks.

In this manuscript, the top100 publications based on high citations and 10 keywords are explored. The selected manuscripts are state-of-the-art models in terms of MEH systems for low-power battery-less IoT devices data extracting, simulation modelling, parameter optimization, enhancement of algorithm, mathematical modelling, simulation and prototype implementation of battery-less IoT devices. The analysis covers the advantages, disadvantages, research gaps, contributions, and type of research work.

**Fig. 7:**
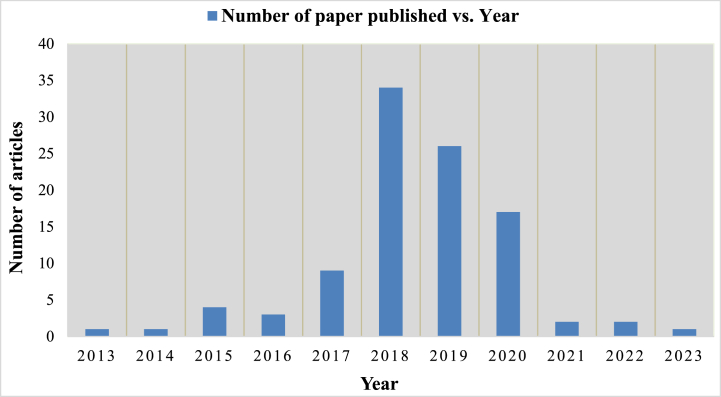
Top 100 most-cited articles distribution (2013–2023).

The distribution of the top 100 articles in the field of MEH system and IoT application between 2013 and 2021 is shown in Fig. 7. It is clear that there was an increase in the number of highly referenced article publishing between 2017 and 2020, reaching a peak of 36 articles in 2018. The number of highly referenced papers then dramatically decreased from 2013 to 2014 and 2023, reaching just 2 in that year. From the analyses of database, the research trends in the MEH system increased from 2015 to 2018 and then there was a gradual decrease from 2019 till 2023. To recognize recent technological advancements and developments in the best algorithm for MEH and IoT applications, we only took into consideration the conference paper with article citations per year (ACYs) was published in year of 2013 accordingly Table 2 data. As a result, while earlier articles had received more citations than more current articles, in terms of average number ACY, the more recent article had the advantage because it addressed the majority of the short-comings and inadequacies in the earlier works.

### 3.5 Bibliometric analysis co-relations of keywords

**Fig. 8:**
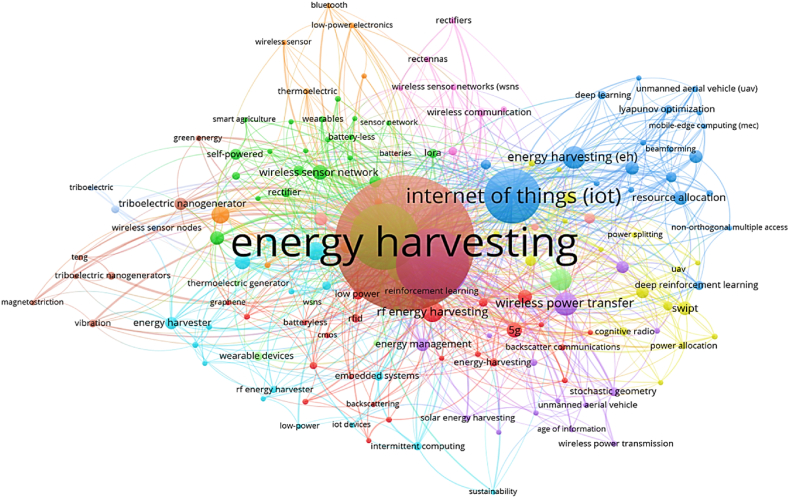
Famous keywords in MEH based IoT devices applications.

The top 100 papers selected from the largest database, Scopus, are shown in Fig. 8. The network of connections between all the keywords in the graphic was created using the software VOSviewer. The circle and its name demonstrate the keywords' influence, while the connecting line between the keywords establishes the relationship.

**Fig. 9:**
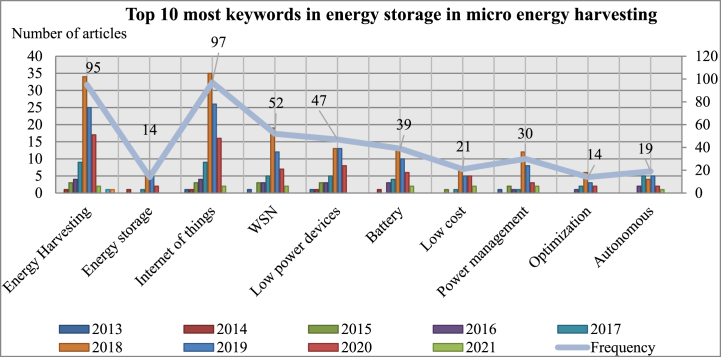
Top 10 keywords distribution over the year 2013–2021.

Fig. 9 is the cluster view of the common keywords in the field of MEH. In Fig. 9 the MEH cluster is linked with different harvesting techniques. The cluster explains power management with devices. The cluster also linked different communication techniques in the IoT platform. Accordingly, in Fig. 8 different colors are used to present various cluster groups by the study's understanding. The brown cluster combines EH resources, including vibration, triboelectric, thermoelectric and solar energy. The blue cluster contains the application of IoT with the MEH system in terms of resource allocation, deep learning, optimization and beamforming techniques. The green cluster highlights the energy power system for IoT applications with and without batteries. This also includes WSNs and electrical equipment like rectifiers. The purple cluster has a significant impact on communication systems such as wireless power transmission, energy management, radio frequency and wireless communication. The red cluster emphasizes low power EH, wireless power transfer, imbedded systems, 5G communication and IoT devices.

The top 10 most popular keywords from the chosen articles from 2013 to 2021 are shown in Table 2. The primary goal of this analysis is to identify current trends in the study of the best algorithm for MEH in IoT applications. Energy harvesting, IoT, WSN and battery-less are the four most frequently used keywords. 97, 95, 52 and 39 are the respective numbers. In recent years, "low power devices," "Low Cost," “Power management”, “Autonomous” and "Optimization," which are interconnected, have received increased attention. In Fig. 9, the top 10 keywords listed in Table 2 are visually illustrated in detail.

**Fig. 10:**
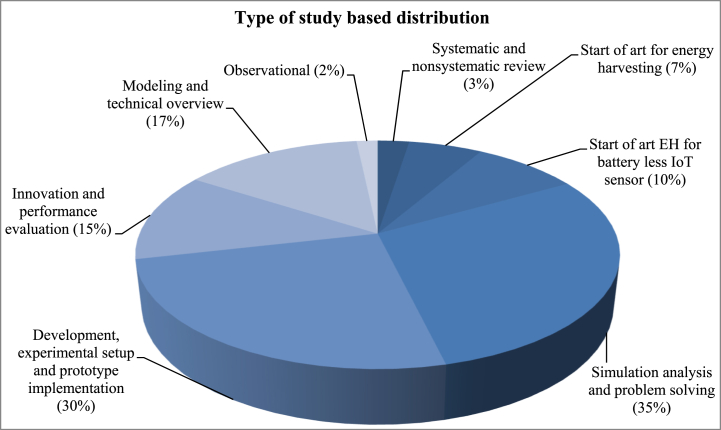
Study type distribution of manuscripts.

In Table 3 there is a correlation between the frequency of publications and year range. It is clear from Table 3 that the articles published in the years 2013–2021 are mostly research-based in the field of IoT, MEH systems, power management, low power devices. Fig. 6 represents the type of research. Fig. 10 clear that in the years 2013–2021 modeling and technical overviews only 17%. The Simulation analysis and problem-solving-based manuscripts are 35% whereas Development, experimental setup and prototype-based manuscripts are 20%.

**Table 3:** Classification of a manuscript based on the type of research.

Research	Number of Publication	Years Limit	Citation Limit
Micro energy harvesting system for low power battery-less IoT devices data extracting, simulation modeling, parameter optimization.	105	2013–2023	59–756
Enhancement of algorithm, Mathematical modeling, simulation and prototype implementation of battery-less IoT devices	100	2014–2023	59–179
Optimization methods for sizing, controls in low powered micro electronic devices, autonomous sensors and power management in low-power IoT devices.	57	2015–2023	59–512
Critical, state-of-the-art, managerial, and technological survey reviews of MEH.	37	2018–2023	59–542
A systematic review based on energy storage, autonomous sensors for wireless sensor network.	17	2019–2023	59–412

Table 4 shows the top ten most-cited publications in low-cost MEH over the last 5 years, as measured by the average citation per year (ACY) and citation rank parameters. It is clear from Table 4 that Van et al. have the highest citations in the last 5 years with 403 ACY. On the other hand, Ejaz et al. have the second highest of 62 number citations; Min et al. with 59 have the third-highest number of citations. In Table 4, the advantages, disadvantages, research gaps, contributions, and type of research work are also highlighted.

**Table 4:** Top ten articles based on “highest citation in the last 5 years.

Rank	References	Last 5 years' citation	Total citation rank	Advantage	Contribution	Research gap	Simulation and experimental study
1	[27]	475	2	Effective in low-power IoT and sensor network communication.	Review of backtrace communication challenges and solutions.	The interference problem should be discussed in detail.	–
2	[30]	400	5	Progress of Textile IoT based electronic devices in last five years is discussed.	Textile integrated conventional IoT device.	Limitations of smart textiles	–
3	[29]	384	4	Investigation of communication offloading in IoT wireless communication	For an IoT device to obtain the best offloading performance without being aware of the MEC model, the energy consumption model, or the computation delay model, a "hot booting" Q-learning based offloading strategy is presented.	RF based offloading scheme.	Simulation based Study.
4	[32]	334	7	The development of flexible PVDF-based piezoelectric sensors and nano-generators is summarized in this study, with particular attention paid to the materials used and their inclusion, the manufacturing process, structural design, and energy harvesting.	For the design of advanced flexible piezoelectric PVDF based nano-generators, a few current difficulties and potential future developments are finally described and explored.	Safety issues.	Study
5	[33]	284	8	With an emphasis on characterization, manufacturing, modelling and simulation, durability and dependability, state-of-the-art harvesting materials and structures are described.	Comparison of modelling and simulation results for three energy harvesting techniques	Influence of electromechanical loading.	Simulation
6	[28]	261	3	Four types of energy harvesting methods namely vibration, light, and thermal energy extraction, wireless energy harvesting (WEH).	Considering their energy efficiency, usability, solution complexity, and Internet connectivity capability, the four designed systems' suitability for extract energy, implementing monitoring applications was examined.	Limitation of WEH low energy devices.	Simulation
7	[31]	239	6	Energy optimization and scheduling for IoT in small cities.	Target efficient energy scheduling and wireless power transfer in IoT devices.	Complexity of surety protocols should be discussed.	Simulation based Study.
8	[35]	214	11	Design of universal and sustainable energy source for on-body electronics.	Design of efficient Triboelectric nano-generator from biomedical energy resources.	Fabricated TENG unitability at higher loads.	Simulation and experimental based study.
9	[36]	214	12	Thermal energy harvesting is 90% more efficient in low energy harvesting.	Recent advancements in the field of thermoelectricity are reviewed, providing an up-to-date comparison and evaluation. These advancements are primarily attributable to multidisciplinary optimization of materials, topologies, and regulating circuits.	Power challenges like: below 20m-V power requirement of controller.	Study based.
10	[21]	199	9	Presents a wearable sensor node that can execute an autonomous WBAN and has Bluetooth low energy transfer and solar energy harvesting.	IoT based smart phone application is designed for health monitoring.	Solar sensor need to be placed outside for short time per day.	Simulation and experimental based.

### 3.6 Evaluation of publisher, country and journal impact factor and review duration

**Fig. 11:**
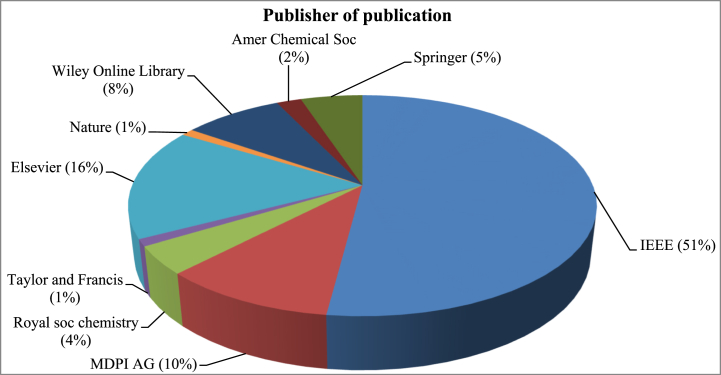
Distribution of articles based on the publisher.

Between the chosen articles, IEEE and Elsevier published 67% of the manuscripts within the designed highest cited articles. Most of the articles were published by IEEE (51%) whereas 16% were published in Elsevier. Among the rest of the 10% articles were published by the Multidisciplinary Digital Publishing Institute (MDPI), 9% in Wiley Online Library, 4% in Royal soc chemistry and 1% in Taylor and Francis. Fig. 11 demonstrates the different publisher's charts where the top-most 100 cited articles were published.

### 3.7 Analysis of main author Contributions

**Fig. 12:**
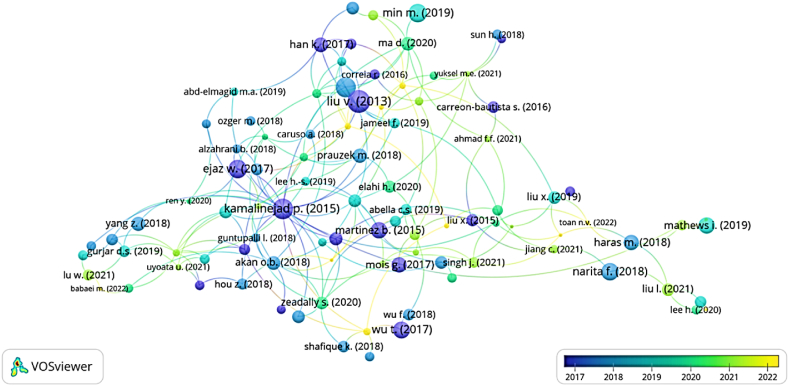
Network mapping of authors with high citation.

The co-citation analysis of the top-cited publications from the Scopus database is shown in Fig. 12. When a database is needed as an input parameter for the keyword analysis, which is created through hand selection of the selected manuscript, VOSviewer software is employed. Five distinct sets of clusters were created from the 100 highly referenced article databases that were chosen, and these are shown in Fig. 12 with different colour codes. In the blue cluster, a strong link between Liv. v, Kamalinejad and Ejaz where focus on low power battery-less IoT devices in the field of micro EH [28,31]. Narita f. and Haris m. are the most well-known authors in the seagreen cluster, and the major goal of their research is to model and simulate berryless systems for IoT applications [33]. Elahi h., Leeh s., and Abella c. s. were discovered to have a strong association and conduct related types of research in the green cluster for the 2019–2020 academic year. Elahi focused on IoT, EH and ambient energy harvesting and Abella highlighted IoT, EH, Low power sensors, storage and autonomous WSN systems [55]. For yellow cluster IoT, WSN, power management, automation and converter are the famous keywords.

### 3.8 Bibliometric analysis co-relations of authors with country

**Fig. 13:**
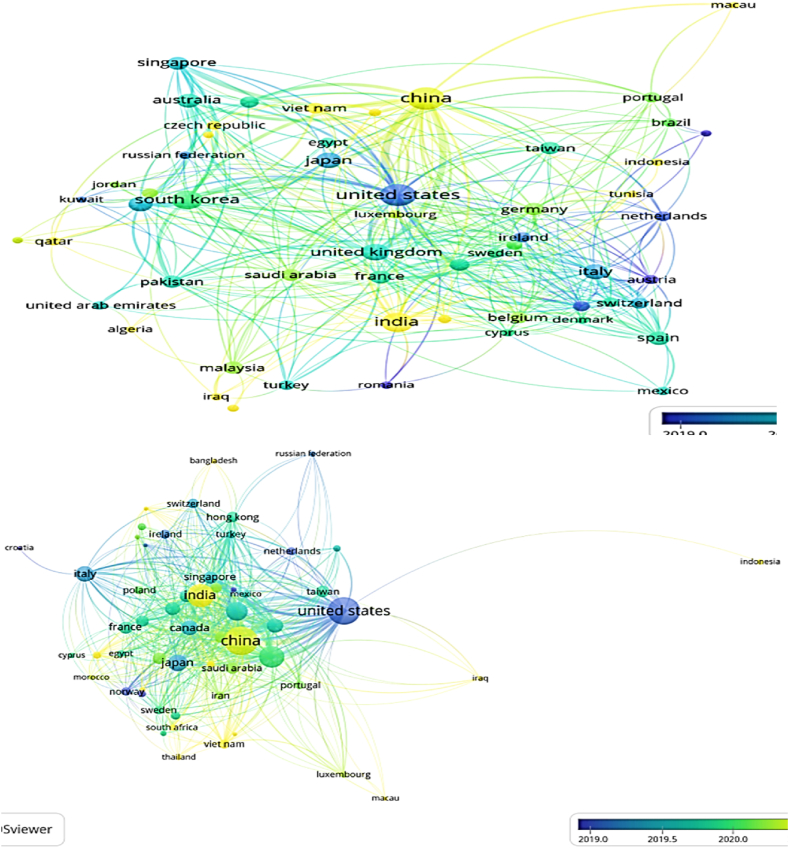
VOSviewer analysis authors with country and citation.

Although condition monitoring research is carried out globally, a few nations stand out as playing key roles. As noted in Table 3, the research done in the United States is distinguished by having the most notable number of publications and citations (in addition, as can be seen in Fig. 13, the United States is distinguished by having the highest strength of interests, as demonstrated by its having the highest diameter circle). According to the quantity of publications, the following nations are: China, India, Australia, South Korea, and Italy. Taiwan, Malaysia, Turkey, Spain, and France are positioned after a number of other nations when the quantity of citations is taken into account. In most of the countries the key research topics are micro energy harvesting, low power IoT applications.

## 4 State of art MEH system for IoT devices

This section critically examines the different technical facets of EH, highlighting its essential elements, features, algorithms, techniques, methodologies, and control strategies in IoT applications.

### 4.1 MEH different techniques and technologies for IoT devices

Over the past ten years, scientific study has focused heavily on EH techniques, which primarily transform ambient energy sources like triboelectric, solar, thermal, electromagnetic, and vibrational energies into electrical forms for powering electronic devices. They are regarded as enabling technology that gives power to IoT devices [125,126]. MEH system a type of EH technique, can use high-performance energy converters for small-scale users. The MEH have several uses in electronic equipment because of appropriate energy density, size flexibility, and simple structure. The main challenges in designing the MEH system relate to the electrical circuit used to create a power management system. When a power management system is created for MEH system, numerous issues must be taken into consideration, including impedance matching, voltage regulation, and electronic components. Another interesting topic to improve system effectiveness is the best geometry for electromagnetic harvesters.

#### 4.1.1 Triboelectric energy harvesting

Triboelectric enables nanogenerators to convert mechanical energy into electric power. Due to their numerous flexible/stretchable configurations, no material restrictions, and high output performance, triboelectric nanogenerators have emerged as a promising EH technology for IoT devices in recent years [127]. They also effectively convert mechanical stimuli from the environment into electricity. Triboelectric nanogenerators can also actively work as self-powered sensors and actuators to detect, monitor, interact with, and respond to ambient changes caused by the environment or humans. These capabilities can be essential for achieving sustainable functional systems. To achieve self-powered, adaptable, and intelligent functional systems, triboelectric nanogenerators are therefore used to wearable IoT electronics, resulting in a highly advanced technology [128].

#### 4.1.2 Solar micro energy harvesting

In [129], the authors designed a brand-new dual-axis solar tracker that can be used to monitor and boost the performance of solar panels. To maximize the output power of the solar panel, this tracker uses a microprocessor to control the movement of the solar panel. The system is created with a straightforward construction and is wirelessly capable, enabling remote monitoring and control with the aid of IoT. To transport the energy stored in supercapacitors from solar cells to drive sensor nodes [130], designed the circuit. The proposed design was made for the micro-watt power levels of indoor light gathering. In tests, it was demonstrated that the configuration could power a wireless temperature and humidity sensor node.

#### 4.1.3 Wind energy

In low power, IoT electronic devices a supercapacitor-based hybrid control system is a good option to store energy produced by low-voltage wind turbines [131]. The authors in Ref. [131] discussed a control system, which used switching technology based on metal-oxide-semiconductor field effect transistors (MOSFETs), allowed the supercapacitor bank to be individually charged from the turbine and subsequently discharged through the battery. In low wind locations, this off-grid method could charge a 6 V or 12 V DC battery even if the turbine's output was only 4 V. The design of a an optimal hybrid renewable energy system is presented by authors in Ref. [132] discussing the hybrid combination of solar and wind generators with batteries and converters. The sensitivity analysis of hybrid systems was also covered in the study, which may be used to assess the impact of uncertainty or a change in a variable and identify the hybrid system's best course of action.

#### 4.1.4 Piezo energy harvesting

Piezo energy harvesting is very popular these days to empower low-powered IoT devices. The authors in Ref. [133] developed an EH structure similar in shape to tree branches. The laminated PVDF beam of model LDT4-028 K/L provided a greater output voltage than the non-laminated beam. Due to the low output power, the generated electricity was first stored in a rechargeable battery before being connected to electrical loads. But to improve its overall performance and make it a stand-alone system, a control circuit is necessary. The authors in Ref. [134] designed a piezo energy harvester that harvests energy from heel strike and toe-off. Two different piezo sensor materials, PVDF and PZT were utilized to compare the outcomes after piezo transducer was implanted in a shoe's insole. On the base of experimental results, the PZT was able to produce up to 150V with 80 mW of electricity, PVDF could only create a maximum voltage of 60V with 20 mW of power. The authors in Ref. [135] discussed a triboelectric MEH for agricultural farm automation. The main disadvantage of a charge amplifier is the high input impedance and temperature dependency. For better results synchronous boost converter would be a better option. The authors in Ref. [136] proposed a machine-learning algorithm for data monitoring and fault detection. The main disadvantage is the time taken by the algorithm to interpret the final model. The performance of the algorithm can be improved by expanding the training data, raising the maximum leaf nodes, adjusting learning rates, and regularization approaches. Table 5 discusses different MEH technologies with their merits and limitations empowering IoT devices. Different MEH techniques have their advantages and limitations.

**Table 5:** Recent research trends empower IoT devices with different abundant energy technologies.

Energy Harvesting Techniques	Goal of research	Design	Performance test	efficiency	Limitation	Reference
Triboelectric	Provide power source for countless sensors	Design of electro-spun nanofiber self-powered triboelectric sensor.	Efficiency of smart monitoring and transmitting data	Efficiency is enhanced by charge amplifier in power management circuit	Fast response and high sensitivity is a challenge	[135]
	Converts the rotational energy into electric power	A linear and rotational mechanical sensor is designed	Performance test in nine different motion modes	97%	Design of stator and mover for the harvester is difficult, the selection of material is important	[137]
	Conversion of hydro energy into electrical energy	Design of a rotational motor with the flow of water	Light emitting diodes connected and observed as load	Efficient design	In the design the selection of a number of plates for high contact separation frequency of motion	[138]
Solar Energy	Power for IoT Devices in Remote Solar Farms	Design of smart management device	Analysis of software and algorithms for managing and controlling individual solar panels	Improved solar array efficiency	Efficiency with robustness is difficult to achieve	[139]
	machine learning for power data monitoring and fault detection in IoT based system	Decision Trees with logic gradient boosting algorithms is employed	Error identification with stored data analysis	12.8% more efficient than the existing systems	Fault detection and maintenance is difficult	[136]
	Wireless remote sensing system for IoT	Design of charge controller to control the battery charging levels	Health monitoring	The efficiency of the system is enhanced by improving battery life	The number of solar panels should be reduced	[140]
Wind Energy	Power source design from wind energy extraction	Harvester extracting the wind speed and electromagnetic energy is designed	Performance varies with wind speed	At a wind speed of 12 m/s peak voltage of 47.4 V is achieved	Output is very low at low wind speed	[141]
		Excellent design of phase side and rotor side converter	Minimize the effect of voltage sag riding	97.88%	Wind energy conversion variation with wind speed	[142]
	IoT devices power source and monitoring in wind farm	Smart monitoring panel for the panel is designed	IoT based smart monitoring panel for wind farming	Human interface from far locations.	Control of wind parameters is complex	[143]
Piezoelectric	Battery less IoT system	See saw structured based harvester is designed	Mechanical response of the system is observes by tip displacement and stress measurement	23.3% better can cantilever beam harvester	Maximum power extraction in wide band is difficult	[144]
	Battery less IoT node	Rectangular cantilever beam design	High resonance with constant displacement	efficient	Difficult to design piezoelectric wireless switch	[145]
	Hybrid nano generator	Tree shaped hybrid piezoelectric energy harvester	Excellent perform ace in open circuit, short circuit and load analysis	95.37%	Piezo output is low even 10 sheets of piezo cannot produce voltage equal to one PV cell.	[133]

### 4.2 IoT devices for EH applications efficiency, power loss, costing

The collaborative nature of IoT offers several benefits, including self-organization, quick deployment, flexibility, and built-in intelligent processing. IoT technology demands constant electricity, which is one factor in the high IoT power usage [146,147]. IoT devices are typically powered by batteries, which sharply reduces their working lifetime. For data exchange the IoT sensors need power. The main limitation of these devices is the maintenance and power source of these huge data sets. Massive IoT refers to the ongoing collecting of vast amounts of data via sensors [148]. As a result, self-adaptive AI-based algorithms are needed to aggregate, assess, and fully comprehend all program objects. Due to the rise of massive datasets and energy-hungry IoT devices, proper energy management is essential [149].

#### 4.2.1 Validation under different operating conditions

The validation of the MEH from different abundant energy resources for low-powered IoT electronic devices under different case studies and operating conditions is discussed in Table 6. The authors in Ref. [150] proposed an RF MEH system and the energy consumption of the IoT devices is adjusted by the selection of power supply duration and duty cycle. WSN, which serve as the IoT fundamental information acquisition system, are made up of a large number of battery-powered wireless micro sensors. The main limitation of the system is the amount of energy that can be stored in battery which is limited. The sensor will be unable to gather data for the sensor network after the battery energy is depleted [151]. In Ref. [152] the authors developed a battery less IoT system for smart sensing of data in wireless sensor nodes and IoT microelectronic devices. The main limitation in the design is to get maximum output at different speeds of the vehicles. In Ref. [153] the authors discussed a bimorph piezoelectric cantilever beam empowering passive infrared sensor in IoT system. The limitation of the piezo harvester is the frequency dependency which can be overcome by matching the frequency of vibrational source with that of harvester beam structure. From Table 6 it is clear that the hybrid EH has the maximum efficiency among different harvesting techniques whereas the piezo energy harvesting has low cost of construction, for agricultural automation bio fuels and solar EH are better options. Table 7 represents the impact of different algorithms in IoT based MEH systems, verified with network model results and reduction in energy consumption with different switching strategies and optimization techniques. Furthermore, the strengths and weaknesses of MEH based IoT systems with different algorithms are also discussed. In Ref. [154] the authors compared different machine learning (ML) based energy saving strategies for IoT devices and analysed that with the application of ML, the system the system ability to monitor the changes in system parameters enhances with reduction in the overall energy consumption of the system. The system efficiency can be improved by power management circuit.

**Table 6:** IoT based MEH with different validation approaches and research limitations.

Ref.	Area of Research	MEH Type	Contributions of the research work	Research Limitations	Validated Approach	Performance
[51]	Architecture design of energy harvester	Abundant energy harvesting	Discussed recently put out design ideas for EH systems, distribution strategies, storage technologies, and control units.	Limited life time of batteries.	Continuous and reliable delivery of power to distributed IoT network of lightweight, scalable, low-storage nodes.	Efficient
[150]	IoT devices RF energy management	Radio frequency energy harvesting	Error measurement by math processing error method	Full access control is difficult to achieve	Prioritize the devices for power supply duration, adjustment of duty cycle while enabling low energy devices to gather energy in the interim.	79%
[152]	Piezoelectric micro energy harvesting	Piezoelectric vibrational EH	Captures the vibrations in the cities	Design of wheel's vibrational energy harvester	Piezoelectric energy harvesting system	Cost effective and efficient.
[153]	Capacitor as power source for low powered devices	Piezoelectric vibrational energy harvesting	Bimorph piezoelectric cantilever beam empowering passive infrared sensor.	Efficiency of the system is frequency dependent and reduces with the frequency.	A bimorph piezo electric capacitor charger is designed at its resonance frequency.	51.4%
[155]	Hybrid (RF and Thermal) MEH with multi source	Radio frequency and thermal EH	Ubiquitous and continuous availability of IoT power source	Designing of synergistic multi source MEH is difficult.	Thermal energy from temperature swings over the day and the radio frequency energy from cell phone towers are suitable.	80%
[156]	Low power WAN (LPWAN) devices in IoT system	Solar EH for low voltage IoT devices	To run LPWAN IoT devices battery-free, extend their useful lives, and boost ecosystem communication effectiveness.	Generated power is low and depends upon sun light availability	Solar-power charged supercapacitor as energy source for LPWAN IoT device	Energy efficient system
[157]	Energy management in IoT devices	Wireless abundant energy harvesting	A switching strategy is adopted with which the sensor nodes perform task with low power consumption	Transmission of sensors collected data with low power.	Balance between the network performance and life time of the IoT network	Efficiently reduces the network transmission delay with low power consumption
[158]	AI based energy harvesting	Bio fuel cells energy harvesting	Addressed primary technological challenges, including a process for ensuring data quality, a way to model the farm's data using business logic, and a comparison of machine learning algorithms.	Processing small files without affecting the performance of the calculation in a large data environment.	Machine learning algorithms	Provides the highest level of accuracy
[159]	Hybrid energy harvesting	Hybrid of solar, thermal, electromagnetic and kinetic energy harvesting	The multiple substrates are integrated into a single, compact platform using a 3-D platform.	Inefficient dynamic evolution of load factors	Small form factor, Smaller parasitic connection impedances which results in increased power efficiency.	Up to 85%
[18]	IoT vibrational energy harvesting	Vibrational energy harvesting	Zero-power energy-autonomous technologies	Design of harvester which can vibrate at wider range of frequency and selection of the material.	Vibrational energy harvesting over wider range of frequencies.	Efficient and cost effective

**Table 7:** Algorithm based low power consumption IoT system.

Research Focus	Algorithm	Goal	Network Model	Strength	Weakness	Achievement	Ref.
Power Management.	Adaptive threshold energy management algorithm.	Transmitting vital information delay is decreased, making nodes capable of high relevance data with higher possibility of transmission.	Nodes of the WSN for the oil and gas pipeline are deployed in clusters, and efficiently reduces the energy consumption for inter-node transmission.	Unique switching strategy and cluster head selection.	Efficient for low power systems.	Manages the energy consumption efficiently with the extension of network lifetime.	[157]
Energy management of IoT based industrial systems.	Generalized Policy Elimination (GPE) algorithm.	Non-orthogonal multiple data access technology.	Bilateral matching model between users and sub-channels created with low-complexity channel resource allocation technique.	Higher efficiency with optimized energy consumption.	Reduction in the quality of transmitted data due to power saving.	Better system's average energy efficiency to that of the non-cooperative centralized scheduling.	[160]
IoT devices energy and time saving.	Joint optimization algorithm.	Overall energy and time saving of the system.	Cluster head can smartly advise the UAV about its time of next arrival.	Reduces system no of bit errors (BER) gradually	Energy efficiency of the system decreases with α and ẞ parameters of the system.	Enhancing the overall efficiency of the system	[161]
IoT with machine learning.	IoT demand side energy management.	Cost effective energy saving for IoT based machine learning system.	The article examines at a number of energy-saving strategies, such as management, technologies, and policy-based energy reductions.	A wide variety of management and decision-making issues can be quickly resolved with low energy consumption via ML.	Design of numerous intelligent energy management systems is challenging.	By using ML to improve energy systems' ability to identify and react to changes in power circumstances, energy waste can be decreased.	[154]
Performance enhancement of IoT system.	Honey badger algorithm.	To maximize the performance of the IoT networks using various networks like vehicular and hoc networks (VANET), wireless body area network (WBAN), mobile ad-hoc network (MANET), radio frequency identification system (RFID) and WSN.	Designing deep learning model called O-RNN-based (deep learning neural network) performance prediction model of IoT for smart city applications.	With the aid of restrictions such energy consumption, data size for both gathered and sent data, mobility, false positives, throughput, packet loss, and latency, the efficiency of each network is predicted.	Simulation of different networks is time taking.	Predicted and increased superiority	[162]
Energy efficient agricultural IoT system.	Shortest route less cost algorithm.	Performance enhancement of IoT-based sustainable applications in a real-time environment.	Model of IoT-based Agriculture Network (IoTAN) simulated and split into regions to enhance the efficiency.	Shortest route for data transmission with less energy combustion.	Improvement of the devices' sustainability without stressing them above their threshold is difficult.	Low cost and energy saving	[163]
Efficient job scheduling for energy management.	Sparrow search algorithm.	Minimization of energy consumption through the efficient task placement.	Balances the demand and reduces resource loss	Efficient resource forming	Requires GEO distributed mobility support.	Low latency and load balance	[164]
Energy management in remote IoT applications.	Deep Q network-based flow work scheduling algorithm.	Low energy consumption at higher data transfer.	Model an intelligent edge-cloud collaboration strategy that uses less energy and produces enough data processing performance.	Reduces energy consumption and cost.	Unstable data path reduces the efficiency.	Data processing between cloud and IoT at low energy	[165]
Energy optimization in ML based IoT system.	Tiny Machine Learning algorithm.	Distant inference in bright environments for an efficient cloud-based model.	machine learning model for battery less IoT	Better accuracy of cloud based system.	Difficult to create conventional natural network on cloud.	Capacitor as power storage device	[166]

## 5 Challenges and suggestions

The MEH is a promising technology that can empower IoT devices without the need for a battery or external power source. However, there are several issues associated with MEH for IoT devices. Some of the key issues are:

### 5.1 Power density

In [167], the authors focused on MEH and indicated that MEH have poor power density which might not be sufficient to power IoT devices. Power density is one of the key issues associated with MEH for IoT devices [168]. In Ref. [168]*,* the authors reported power density as the amount of power that can be generated per unit of volume or weight of the EH. The power density is an important parameter in the design of MEH for IoT devices [169]. The first challenge is that the output power in the MEH is usually very low. For instance, solar energy has a relatively low power density, and inside illumination has an even lower EH potential. Accordingly, to provide enough power to run IOT devices, energy harvesters must be extremely efficient [170]. Second, the size of the EH can also impact its power density. The power density of a large EH is usually more than that of a smaller EH. This is because the size of the EH directly affects the amount of energy that can be extracted. Nevertheless, to be integrated into IOT devices, the size of EH can be reduced, which can further lower their power density.

Lastly, the power density of an EH can also be affected by the technology employed for energy harvesting. The features of power density vary amongst EH types. Thermoelectric EH can generate low power density but are better suited for low-grade heat sources, whereas piezoelectric EH can produce high power density but require high mechanical stress [171]. Current research and improvement efforts are concentrated on enhancing the power output and efficiency of EH. To creating a new type of EH that can generate higher power densities in order to address the problem of low power density for MEH for IoT devices. Furthermore, harvested energy can be stored and used to provide a more reliable power source for IoT devices through the use of energy storage technologies like supercapacitors and rechargeable batteries [156].

### 5.2 Efficiency

Efficiency is an important issue associated with MEH system for IoT devices [150]. The ability of the harvester to transform the ambient energy into useful electrical energy is referred to as efficiency of the EH. The majority of EH devices have low efficiency levels, which means that a significant amount of energy is lost during the energy conversion process which may therefore restrict the functionality of IoT devices.

The effectiveness of MEH can be impacted by various factors. First, the design of the energy harvester can impact its efficiency. The energy harvester's efficiency can be affected by its components, its geometry, and the technique to convert the energy [172]. Furthermore, the energy source itself may affect the energy harvester's efficiency.

Second, the EH efficiency may also be impacted by its operating circumstances. The efficiency of the EH can be decreased and its performance is affected by temperature, humidity, and other environmental factors [173]. Because of these operating circumstances, it may be challenging to estimate how much energy can be obtained from ambient sources. The efficiency of MEH for IOT devices was discussed by the authors in Ref. [173]. Current research and development efforts are concentrated on enhancing the EH functionality and design. The research also focused on new harvesting materials, innovative production techniques, and innovative circuit designs that can raise energy conversion efficiency.

### 5.3 Size and form factor

For EH to be combined into IoT devices, they must be compact and light [174]. However, size and factors can be challenging issues in design. Size and form factors are critical issues associated with MEH for IoT devices [175]. IoT devices are typically small and have limited space for integrating EH. Energy harvesters must therefore be made smaller to be incorporated into IOT devices. Nevertheless, size reduction can have a poor effect on EH power density and efficiency, making it tough to generate enough power to run IoT devices [18]. There are numerous factors which can effect the size and form factor of MEH. First, the size and form factor of an energy harvester can be affected by its harvesting technology. For instance, thermoelectric EH is larger and more rigid than piezoelectric EH, which is usually thin and flexible.

Second, the EH dimensions and form factor may be affected by the materials it is made of. Materials that are lightweight and flexible can enable EH to be more easily integrated into IoT devices. However, lightweight and flexible materials can also impact the durability and performance of the EH. Third, the EH dimensions and form factor may be affected by the production method. Energy harvesters can be made smaller and less expensive by utilizing scalable manufacturing processes and that can power large numbers of devices.

In order to tackle the problem of form factor and size for MEH in IoT devices, current research trends are focussed on the development of EH that are scalable, lightweight, and highly efficient. This involves creating novel materials and production techniques that will make it possible to produce energy harvesters with more compact form factors with better functionality and durability of the devices.

### 5.4 Cost

Cost is an important issue associated with MEH for IoT devices [176]. The materials and manufacturing processes used to create energy harvesters can be expensive, which can increase the cost of the IoT device. The authors in Ref. [176] reported energy harvesters can be expensive to produce, and the cost can impact the commercial viability of IoT devices. The expense of EH can effect the scalability of IoT devices, particularly for large-scale applications.

Different parameters can effect the expense of MEH. First, the materials used in the EH can impact its cost. Materials that are rare or difficult to produce can increase the cost of energy harvesters [177]. Additionally, the cost of manufacturing energy harvesters can impact their overall cost. Manufacturing processes that are complex or require specialized equipment can increase the cost of energy harvesters.

Second, the EH cost may vary depending on its dimensions and form factor. Because they require specialized materials and manufacturing techniques, sometimes the cost of small EH is higher. Ongoing research and development efforts are concentrated on creating affordable EH to address the issue of cost for MEH for IoT devices [178]. This covers the creation of novel, affordable materials and scalable manufacturing techniques. Furthermore, research is concentrated on creating EH that can be inexpensively and widely produced in large quantities while being integrated into current manufacturing processes.

Optimizing IoT devices' energy management systems to reduce their energy consumption is another strategy for resolving the cost issue. The EH can be made smaller and more affordable, and it can function more effectively, by lowering the power losses. This includes the creation of low-power modes and power management strategies that can increase a device's battery life and lower its energy needs.

### 5.5 Integration

Integration is a critical issue associated with MEH for IoT devices [179]. The study in Ref. [179] addressed energy harvesters need to be integrated seamlessly into IoT devices to provide a reliable and sustainable power source. Nevertheless, integrating EH into IoT devices can be challenging due to the complex and varied nature of IoT devices. The integration of MEH into IOT devices can be impacted by a number of variables. Initially, the EH form factor may affect how well it integrates with the IOT device. Energy harvesters must be made to match the unique specifications of IOT devices, such as their dimensions and form, as well as where they should be placed inside them.

Second, the EH output must match the IOT device's power specifications. The EH must be able to produce enough power to meet the power needs of various IOT devices, each of which has different power requirements. Furthermore, the EH voltage and current output must work with the IOT device's power management system. Thirdly, the energy harvester integration must work with the IoT device's manufacturing process. The energy harvester must be made to be easily integrated into the production process without compromising the IoT device's durability or functionality.

Research and development efforts are being directed toward creating EH that are simple to integrate into IOT devices in order to address the problem of integration for MEH for IoT devices [180]. This includes creating EH that work with a variety of IOT devices and are simple to modify to fit each one's unique needs. Furthermore, research is concentrated on creating methods to maximize the energy output of energy harvesters by strategically placing and orienting them within IoT devices. Finally, the development of standard interfaces and protocols for energy harvesters can facilitate their integration into IoT devices and improve interoperability between different devices and systems. Here are some suggestions for MEH for IoT devices:

• Investing in emerging MEH technologies and materials that can enhance the stability and effectiveness of energy conversion.
• Improving compatibility between MEH systems and IoT devices by creating standardized MEH interfaces and protocols. Utilizing a comprehensive approach to explore the social and technical challenges of MEH in IoT.
• Solar power is one of the most widely used micro energy harvesting technologies. IoT devices may produce power from the sun's light and consume it to power themselves by integrating small solar panels.
• IoT devices can also generate power by using piezoelectric materials to transform motion into electricity. These materials can be incorporated into the design of the device to produce energy through vibrations or movement.
• Thermoelectric MEH, which involves converting temperature gradients into electrical power using thermoelectric materials, offers promising solutions for powering small electronic devices. However, it also faces several challenges and limitations of low efficiency, low temperature gradient and cost etc. Despite these challenges, ongoing research and development efforts are focused on improving the efficiency and practicality of thermoelectric micro energy harvesting.
• IoT devices have the ability to harvest energy from RF signals in their surrounding area, such as those given off by Wi-Fi routers and cell towers. In areas with weak RF signals, harvesting efficiency may be lower. But the range and coverage of RF energy harvesting depend on the strength of available RF signals. Strengthening RF signals for MEH can be challenging, as it involves capturing and converting existing RF signals from the environment rather than actively transmitting RF power. However, RF energy for EH can be optimized by antenna design, optimized antenna placement, frequency band selection, EH circuit design and source proximity etc.
• IoT devices can run independently for long periods of time without the need for external power sources thanks to MEH, which makes them perfect for applications where power is expensive or challenging to get.

## 6 Conclusions

Citation analysis within a certain field of study and journal is the only reliable indicator of an author's, journal's or article's impact. The status of a citation may indicate the academic significance of an article when all the previously mentioned constraints are considered. The purpose of this study is to list, analyse, and classify the attributes of the top 100 publications in MEH for the IoT platform. A variety of studies have also been presented, such as the distribution of articles by study type and subject matter, the most well-known authors, journals, and publishers, the biblio-metric analysis of the co-occurrence keywords, and the countries that published the most highly cited manuscripts. The study's ultimate objective is to present the performance, efficiency and research gaps of the IoT devices powered by ambient energy resources in the most recent research. The findings discussed the development of MEH with different techniques and technologies for low powered IoT devices. Validation under different operating conditions is discussed in detail. Table 7 shows how various algorithms affect IoT-based MEH systems, as confirmed by network model findings. It also shows how various switching methods and optimization techniques can reduce energy usage. Additionally, the advantages and disadvantages of IoT systems based on MEH and various algorithms are also covered. Several limitations associated with MEH for IoT devices are discussed.

In conclusion, MEH is an important and rapidly evolving field with significant potential for powering IoT devices sustainably and cost-effectively. Despite the limitations, MEH remains a valuable technology for IoT applications, especially in scenarios where the benefits of sustainability, long-term maintenance-free operation, and reduced environmental impact outweigh the limitations. Further research and development are needed to address the current challenges and unlock the full potential of MEH for IoT.

## CRediT authorship contribution statement

**Mahidur R. Sarker:** Writing – review & editing, Writing – original draft. **Amna Riaz:** Writing – review & editing. **M.S. Hossain Lipu:** Investigation, Formal analysis. **Mohamad Hanif Md Saad:** Writing – review & editing, Investigation, Formal analysis. **Mohammad Nazir Ahmad:** Writing – review & editing, Investigation, Formal analysis. **Rabiah Abdul Kadir:** Writing – review & editing, Investigation, Formal analysis. **José Luis Olazagoitia:** Writing – review & editing, Funding acquisition, Formal analysis.

## Declaration of competing interest

The authors declare that they have no known competing financial interests or personal relationships that could have appeared to influence the work reported in this paper.
